# Hypoxia-dependent expression of MAP17 coordinates the Warburg effect to tumor growth in hepatocellular carcinoma

**DOI:** 10.1186/s13046-021-01927-5

**Published:** 2021-04-08

**Authors:** Fangyuan Dong, Rongkun Li, Jiaofeng Wang, Yan Zhang, Jianfeng Yao, Shu-Heng Jiang, Xiaona Hu, Mingxuan Feng, Zhijun Bao

**Affiliations:** 1 Department of Gastroenterology, Huadong Hospital, Shanghai Medical College, Fudan University, No.221 Yan’an West Road, Shanghai, 200040 P.R. China; 2 Shanghai Key Laboratory of Clinical Geriatric Medicine, Shanghai, 200040 P.R. China; 3 Research Center on Aging and Medicine, Fudan University, Shanghai, 200040 P.R. China; 4 Department of Geriatrics, Huadong Hospital, Shanghai Medical College, Fudan University, Shanghai, 200040 P.R. China; 5 Institute of Oncology, Affiliated Hospital of Jiangsu University, Zhenjiang, 212001 China; 6 State Key Laboratory of Oncogenes and Related Genes, Shanghai Cancer Institute, Ren Ji Hospital, School of Medicine, Shanghai Jiao Tong University, Shanghai, 200240 P.R. China; 7 Department of Liver Surgery, Ren Ji Hospital, School of Medicine, Shanghai Jiao Tong University, 1630 Dongfang Road, Shanghai, 200127 P.R. China

**Keywords:** PDZK1IP1, SLC2A1, Aerobic glycolysis, Liver cancer, Hypoxia-inducible factor

## Abstract

**Background:**

Reprogrammed glucose metabolism, also known as the Warburg effect, which is essential for tumor progression, is regarded as a hallmark of cancer. MAP17, a small 17-kDa non-glycosylated membrane protein, is frequently dysregulated in human cancers. However, its role in hepatocellular carcinoma (HCC) remains largely unknown.

**Methods:**

Immunohistochemistry was used to analyze the expression pattern of MAP17 in HCC. Loss-of-function and gain-of-function studies were performed to investigate the oncogenic roles of MAP17 in vitro and in vivo. RNA sequencing, co-immunoprecipitation, immunofluorescence and western blotting were used to study the molecular mechanism of MAP17 affecting the tumor growth and glycolytic phenotype of HCC.

**Results:**

An integrative analysis showed that MAP17, a small 17-kDa non-glycosylated membrane protein, is significantly related to the glycolytic phenotype of hepatocellular carcinoma (HCC). Firstly, we found that MAP17 expression is hypoxia-dependent and predicts a poor prognosis in HCC. Genetic silencing of MAP17 reduced the rate of glucose uptake, lactate release, extracellular acidification rate, and expression of glycolytic genes. Ectopic expression of wild type MAP17 but not its PDZ binding domain mutant MAP17-PDZm increased tumor glycolysis. Further research showed that MAP17 knockdown markedly retarded in vivo tumor growth in HCC. Importantly, attenuation of tumor glycolysis by galactose largely hijacked the growth-promoting role of MAP17 in HCC cells. RNA sequencing analysis revealed that MAP17 knockdown leads to transcriptional changes in the ROS metabolic process, cell surface receptor signaling, cell communication, mitotic cell cycle progression, and regulation of cell differentiation. Mechanistically, MAP17 exerted an increased tumoral phenotype associated with an increase in reactive oxygen species (ROS), which activates downstream effectors AKT and HIF1α to enhance the Warburg effect. In HCC clinical samples, there is a close correlation between MAP17 expression and HIF1α or phosphorated level of AKT.

**Conclusions:**

Our results show that MAP17 is a novel glycolytic regulator, and targeting MAP17/ROS pathway may be an alternative approach for the prevention and treatment of HCC.

**Supplementary Information:**

The online version contains supplementary material available at 10.1186/s13046-021-01927-5.

## Introduction

Hepatocellular carcinoma (HCC) comprises the majority of primary liver cancer and represents a serious international health problem [1]. HCC ranks as the fourth leading cause of cancer-related death globally and is expected to continue to increase in the next two decades. Despite great achievement made in diagnosis and therapy, the 5-year survival rate of HCC patients is still unsatisfactory [2]. In advanced HCC patients, the recurrence rate is approximately 80% [3]. Therefore, elucidating novel molecular mechanisms that can help determine how HCC development and progression are still urgently needed.

The hypoxic tumor microenvironment is a major character of solid tumors, including HCC. To meet the high demand for rapid cell proliferation, cancer cells reprogram their metabolisms. Reprogrammed energy metabolism is emerged as a novel hallmark of cancers, especially aerobic glycolysis, also known as the Warburg effect [4]. Aerobic glycolysis can provide a large number of intermediate products for the synthesis of nucleotides, lipids, and non-essential amino acids, and therefore support the rapid proliferation of tumor cells. As the last step of aerobic glycolysis, the produced lactic acid can acidify the tumor microenvironment, thereby inhibiting the activity of immune cells and promoting tumor invasion and metastasis. ​​At the same time, NADPH and reduced glutathione are produced through pentose phosphate branching, which can help tumor cells avoid oxidative stress-mediated damage [5]. Thus, compared with oxidative phosphorylation in normal cells, tumor cells are more dependent on aerobic glycolysis [5]. In HCC, aerobic glycolysis is widespread and is closely related to tumor occurrence, invasion, and patient prognosis. Blocking aerobic glycolysis can significantly inhibit tumor growth and increase tumor cell sensitivity to chemotherapy drugs [6–8]. Therefore, targeting aerobic glycolysis has extremely high translational significance for the development of new therapeutics in HCC.

Previously, Hypoxia-inducible factors (HIFs, including HIF1α and HIF2α) and several oncogenic proteins (AKT, FOXK1/2, SIX1, and c-MYC) are broadly expressed in human cancers and have been reported to be involved in the regulation of the Warburg effect [9–13]. MAP17 is a small non-glycosylated protein, localized in the cell membrane and Golgi apparatus, and is encoded by the *PDZK1IP1* gene [14]. The hydrophobic N-terminus of MAP17 consists of 13 amino acids and contains a PDZ binding domain and two transmembrane regions. MAP17 can interact with proteins containing PDZ domains such as PDZK1 (NHRF3) and other NHRF proteins (NaPiIIa and NHe3). Through interaction with NHRF3 and NHRF4, MAP17 can lead to the internalization of NaPilla in the trans-Golgi network [15]. By stimulating sodium-dependent glucose transporter (SGLT1/2), MAP17 can increase the sodium-dependent specific transport of mannose and glucose in Xenopus oocytes [16]. In tumors, most studies have shown that MAP17 acts as an oncogene with increased tumorigenicity when it is overexpressed [17–21]. However, the cellular function and molecular mechanism of MAP17 in HCC remain scanty.

In this study, we performed an integrated analysis and identified MAP17 as a hypoxia-induced glycolytic regulator in HCC. By loss-of-function and gain-of-function studies, we revealed that MAP17 couples aerobic glycolysis to tumor growth in HCC via activation of ROS/AKT pathway. Thus, this study indicates that MAP17-mediated cascades may act as a candidate therapeutic target for HCC treatment.

## Materials and methods

### Cell culture

Liver cancer cell lines (SK-H929, HepG2, SMMC-7721, Huh-7, MHCC-97H, and HCC-LM3) used in this study were acquired from the Cell Bank of the Chinese Academy of Sciences (Shanghai, China). Mycoplasma contamination detection and short tandem repeat (STR) profiling were performed before cell experiments. Cells were maintained in a 37 °C humidified incubator with 5% CO_2_ and cultured in Dulbecco’s modified Eagle’s medium (Gibco, 11,965,092) or Roswell Park Memorial Institute 1640 medium (Gibco, 11,875,093), supplemented with 1% (v/v) streptomycin-penicillin (Sigma-Aldrich, Shanghai, China) and 10% fetal bovine serum (FBS, Gibco). To mimic hypoxic conditions, HCC cells were maintained in a low-oxygen chamber (< 1% O_2_) for 24 h. The antioxidant N-acetylcysteine (NAC), galactose, MG132, and AKT inhibitor MK-2206 were all purchased from Selleck (Shanghai, China). CHX was purchased from Sigma-Aldrich (Shanghai, China).

### Online database analysis

The BioGPS database (http://biogps.org/) was used to determine the expression pattern of MAP17 in normal human tissues. The Gene Expression Profiling Interactive Analysis 2 (GEPIA2) database (http://gepia.cancer-pku.cn/index.html) [22] was used to determine the expression landscape of MAP17 across 33 types of human cancers.

### Clinical samples

All HCC tissue samples used in this study were obtained from the Department of Liver Surgery, Ren Ji Hospital, School of Medicine, Shanghai Jiaotong University. An HCC tissue microarray containing 202 matched tumor and nontumor tissues was generated as reported previously [23]. All clinical specimens were obtained with informed consent, and protocols were approved by the ethical review committee of the Ren Ji Hospital, School of Medicine, Shanghai Jiaotong University.

### Immunohistochemistry

Immunohistochemical (IHC) analysis was performed as described previously [24]. The primary antibodies used for IHC analysis were shown as follows: MAP17 (1:200, Abcam, ab156014), p-AKT (1:100, Cell Signaling Technology, #4060), and Ki67 (1:400, Cell Signaling Technology, #9449). Scoring was evaluated independently by two senior pathologists who were blinded to clinicopathologic data and calculated based on the percentage of positive staining cells and staining intensity.

### Generation of stably knockdown cells

Short hairpin RNAs (shRNAs) against MAP17 gene were transfected along with a three plasmid system (pPACKH1-REV, pPACKH1-GAG, and pVSV-G) into HEK293T cells using Lipofectamine 2000 (Invitrogen, Carlsbad, CA, USA) according to the manufacturer’s protocols. Conditioned medium (CM) containing retroviral particles was collected at 48 h after transfection, and filtered through 0.45-μm filters. After incubating with conditioned medium containing retroviral particles and 8 mg/ml polybrene (Sigma-Aldrich, H9268, St. Louis, MO) for two days, the target cells were selected with 2 μg/ml puromycin for 2 weeks to yield shRNA-expressing cells.

### siRNA transfection

Small interfering RNAs (siRNAs) targeting HIF1α (si-HIF1α-1, GAGGAAGAACUAAAUCCAAdTdT; si-HIF1α-2, UGAUACCAACAGUAACCAAdTdT), and negative control siRNA were purchased from GenePharma (Shanghai, China). The full-length MAP17 and its PDZ binding domain mutant MAP17-PDZm plasmids were synthesized by Shanghai Generay Biotech Co., Ltd. (Shanghai, China). HCC cells were transfected with siRNAs using Lipofectamine RNAiMax reagent (Invitrogen, USA) according to the manufacturer’s protocol.

### Real-time quantitative PCR

Total RNA was isolated from HCC cell lines using the RNAiso Plus reagent (Takara, Japan) and reversely transcribed through PrimeScript RT-PCR kit (Takara, Japan). The SYBR green-based real-time reverse transcription-polymerase chain reaction (RT-PCR) assay was used to determine gene expression on a 7500 Real-time PCR system (Applied Biosystems, Inc. USA). The housekeeping gene β-actin was served as an internal control for RNA quantity. Primers used in this study were shown as follows: MAP17 forward, 5′-TGGATGCAGGGCCTTATCG-3′; MAP17 reverse 5′-CGACGGTCAGGATCATGTGT-3′; HK2 forward, 5′-TTGACCAGGAGATTGACATGGG-3′; HK2 reverse 5′-CAACCGCATCAGGACCTCA-3′; ENO1 forward, 5′-TGGTGTCTATCGAAGATCCCTT-3′; ENO1 reverse 5′-CCTTGGCGATCCTCTTTGG-3′; LDHA forward, 5′-ATGGCAACTCTAAAGGATCAGC-3′; LDHA reverse 5′-CCAACCCCAACAACTGTAATCT-3′; β-actin forward, 5′-ACTCGTCATACTCCTGCT-3′, β-actin reverse, 5′-GAAACTACCTTCAACTCC-3′.

### Western blotting

Whole-cell extracts were extracted using RIPA buffer (150 mM NaCl, 50 mM Tris-HCl at pH 7.4, 1 mM EDTA, 0.1% SDS, 1% Triton X-100, 1% sodium deoxycholate and 1% NP-40) mixed with a protease and phosphatase inhibitor cocktail for 15 min on ice. The protein concentration was determined by a BCA Protein Assay Kit (Pierce Biotechnology, USA). Standard western blotting protocol was used and following antibodies were used in this study to analyze protein expression: MAP17 (1:1000, Abcam, ab156014), HIF1α (1: 1000, Abcam, ab113642), p-P38 (1:1000, Cell Signaling Technology, #4511), P38 (1:1000, Cell Signaling Technology, #8690), p-S6K (1:1000, Cell Signaling Technology, #9204), S6K (1:1000, Cell Signaling Technology, #9202), p-Akt (1:2000, Cell Signaling Technology, #4060), Akt (1:1000, Cell Signaling Technology, #4685), p-p44/42 MAPK (1:1000, Cell Signaling Technology, #4370), p44/42 MAPK (1:2000, Cell Signaling Technology, #4695), p-STAT3 (1:1000, Cell Signaling Technology, #9145), STAT3 (1:2000, Cell Signaling Technology, #12640), PDZK1 (1:1000, Santa Cruz Biotechnology, sc-390,964), and β-actin (1:2000, Abcam, ab8226).

### RNA sequencing analysis

To illustrate the molecular mechanism of MAP17 in HCC, RNA sequencing was performed at sh-Ctrl and sh-MAP17 SMMC-7721 and HCC-LM3 cells. Preparation of the RNA library and sequencing were performed by Sinotech Genomics (Shenzhen, China). Gene expression was calculated using the Fragments Per Kilobase Million (FPKM) method. Differentially expressed genes were selected using a fold change expression cut-off of 2 and a *P*-value < 0.05. Differentially expressed genes were further subjected to enrichment analysis by CytoScape software.

### Glucose uptake and lactate production

HCC cells were seeded into a 6-well plate and cultured in a medium containing 4.5 g/L glucose supplemented with 10% FBS under hypoxia or normoxia conditions. After 24 h, the cell culture supernatants were collected and subjected for analysis. Glucose uptake was analyzed using the Amplex Red Glucose/Glucose Oxidase Assay Kit (A22189, Thermo Fisher Scientific, USA). Lactate production in the culture medium was determined by using the Lactate Assay Kit (K607–100, BioVision, USA). All results were normalized to the total protein amounts of cells that were measured by Pierce BCA Protein assay (Pierce Biotechnology, USA).

### Measurement of glycolytic capacity

Extracellular acidification rate (ECAR) was analyzed by the Seahorse Bioscience XF96 Extracellular Flux Analyzer (Seahorse Bioscience, USA) with Seahorse XF Cell Glycolysis Stress Test Kit (Seahorse Bioscience, USA). Briefly, 2 × 10^4^/well HCC cells were seeded into an XF96-well plate and maintained overnight. The next day, cells were incubated with an unbuffered medium followed by sequential injection of 10 mM glucose (Glc), 0.5 μM oligomycin (Oligo), and 80 mM 2-deoxyglucose (2-DG) to measure ECAR. Finally, the ECAR value was normalized to the protein amounts of cells before making comparisons between groups.

### Measurement of ROS levels

For cellular ROS analysis, HCC cells were seeded in black 96-well plates at a density of 2 × 10^4^ cells per well. Then cells were subjected to DCF-DA (10 mmol/L) staining in phenol red-free medium for 30 min. Finally, the fluorescence intensity was measured immediately using a BioTek fluorescence plate reader.

### Measurement of HIF1α activity

To determine the effect of MAP17 on HIF1α transcriptional activity, nuclear extract lysates were obtained from MAP17 knockdown or MAP17-expressing HCC cells by using a Nuclear Extraction Kit (#2900, Millipore). The activities of HIF1α in nuclear extract lysates were detected using the HIF1α Transcription Factor Assay Kit (ab133104, Abcam) according to the manufacturer’s protocols.

### Proximity ligation assay

Proximity ligation assay (PLA, Olink Bioscience, DUO92007) was performed as reported previously [24]. Briefly, SMMC-7721 and HCC-LM3 cells seeded onto IBIDI μ-Dish and fixed with 4% paraformaldehyde, followed by permeabilization using 0.1% Triton-X and blocked with 5% BSA in PBS. Mouse anti-PDZK1 (1:200, Santa Cruz Biotechnology, sc-390,964) and rabbit anti-MAP17 (1:100, Abcam, ab156014) were used as primary antibodies. The next day, cells were incubated with the anti-mouse and anti-rabbit probes. Ligation and amplification reactions were carried out according to the manufacturer’s protocols. Cells were then washed, and the nuclei were stained with DAPI. Finally, the nuclei and proximity ligated foci were visualized on a confocal microscope.

### Co-immunoprecipitation experiment

The supernatants were pre-incubated with protein A/G PLUS agarose (Santa Cruz Biotechnology, sc-2003), and subsequently incubated with 2 μg of MAP17 antibodies or control IgG overnight at 4 °C, followed by addition of 20 μl protein A/G agarose for 2 h at room temperature. After washing three times with lysis buffer, immunoprecipitates were boiled in 1 × loading buffer and subjected for standard western blotting analysis.

### Chromatin immunoprecipitation-PCR

Chromatin Immunoprecipitation (ChIP) experiment was performed in SK-H929, MHCC-97H, and Huh7 cells under 20% O_2_ and 1% O_2_ condition. In brief, HCC cells were fixed with 1% formaldehyde for 10 min at room temperature followed by incubation with 0.125 M glycine for 10 min. Cells were washed with ice-cold PBS for three times. Then, fixed cells were collected and resuspended in lysis buffer, supplemented with protease inhibitor cocktails, and sonicated to obtain chromatin fragments of about 500–1500 bp. The supernatants were collected and incubated overnight with anti-HIF1α antibody (F7425) and Dynabeads Protein G. The beads were washed, and the precipitated chromatin complexes were collected, purified, and de-crosslinked. Finally, the precipitated DNA fragments were analyzed by RT-PCR.

### Plate colony formation assay

HCC cells were counted and seeded in 6-well plates with 500–1000 cells per well. Cells were allowed to grow for 10–14 days and the culture medium was replaced every three days. Finally, the cells were fixed with 4% paraformaldehyde, and colonies were counted after staining with 0.1% crystal violet for 20 min. All the experiments were performed in triplicate and repeated three times independently.

### In vivo tumorigenicity

To generate subcutaneous xenograft, 2 × 10^6^ SMMC-7721 and HCC-LM3 cells stably transfected with sh-MAP17 or sh-Ctrl were suspended in 100 μL PBS and then inoculated subcutaneously into the BALB/c nude mice (male, 6-week old). The volume of xenograft neoplasms was monitored and estimated as follows: tumor volume = length×width^2^/2. At the endpoint of the experiment, the mice were sacrificed and the xenograft neoplasms were harvested and weighed. This study was approved by the Research Ethics Committee of Shanghai Jiao Tong University carried out following the guidelines of the national animal protection and ethics institute.

### Statistical analysis

All the data were presented as means ± SEM. Differences were compared using two-tailed Student’s t-test or one-way ANOVA followed by post hoc Duncan tests. The GraphPad Prism (GraphPad Software Inc., San Diego, CA) was used for statistical analyses. The prognostic analysis was conducted by the Kaplan-Meier method and analyzed by the log-rank test. A *P* value of less than 0.05 was considered statistically significant.

## Results

### Hypoxia-dependent overexpression of MAP17 in HCC

**Fig. 1:**
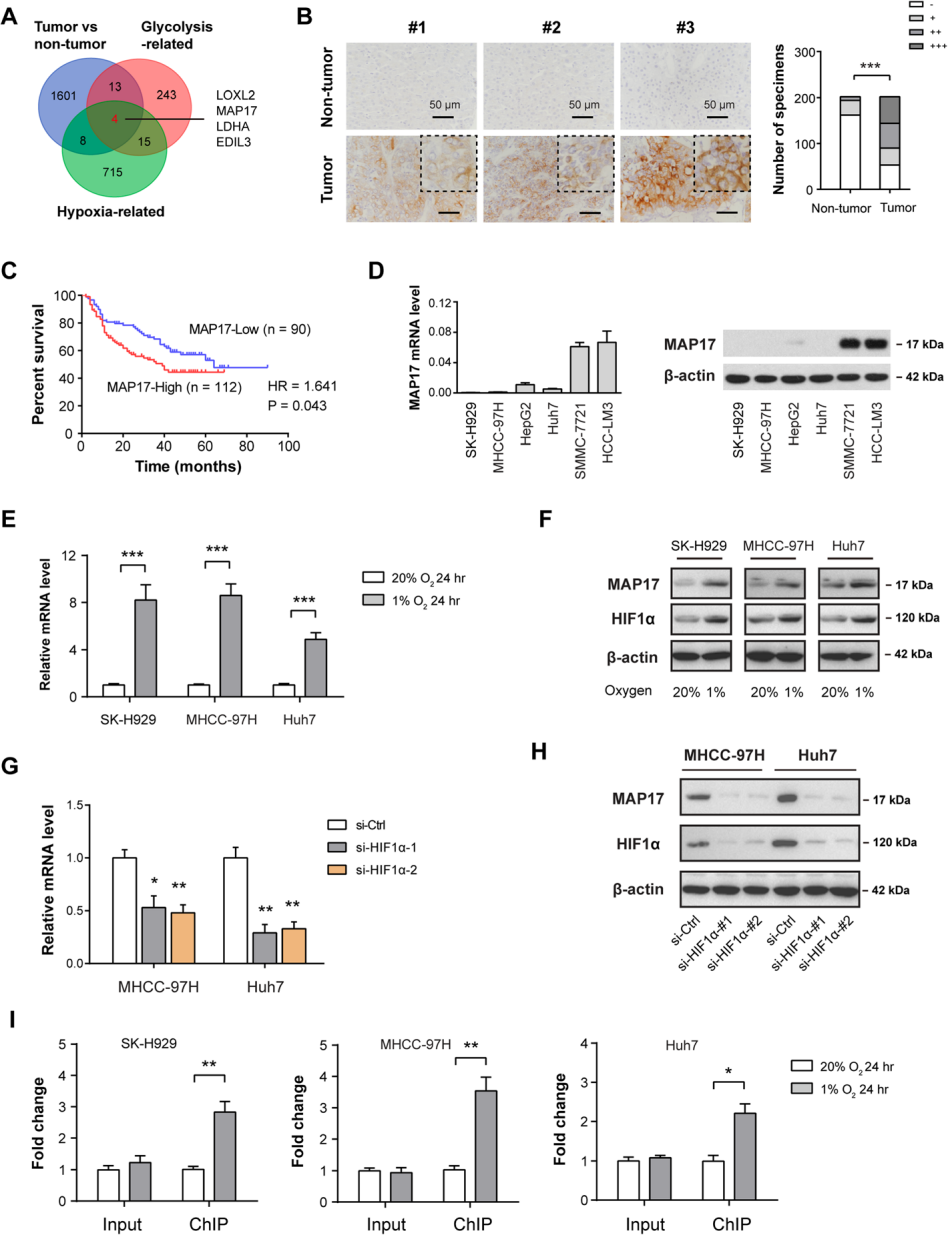
Hypoxia-dependent overexpression of MAP17 in HCC. **a** Venn diagram showed differentially expressed genes (DEGs) related to hypoxia and glycolysis in HCC; The Cancer Genome Atlas (TCGA) cohort (*n* = 369) was used for identifying glycolysis-related genes and hypoxia-related genes in HCC. **b** Immunohistochemical analysis of MAP17 protein expression in 202 matched HCC tissues and nontumor tissues; representative images of MAP17 staining were shown at the left panel; right panel showed the number of specimens displaying high or low MAP17 staining within the tumor or nontumor tissues (Fisher’s exact test, ***P < 0.001); scale bar: 50 μm. **c** Kaplan-Meier curve analysis for overall survival was performed according to MAP17 expression in HCC patients (*n* = 202); HR: hazard ratio. **d** Real-time qPCR and western blotting analysis of MAP17 expression in liver cancer cell lines. **e**-**f** SK-H929, MHCC-97H, and Huh7 cells were cultured under hypoxia (1% O_2_) and normoxia (20% O_2_) for 24 h, followed by detection of MAP17 mRNA expression by real-time qPCR (**e**, n = 3) and western blotting (**f**), respectively. **g**-**h** Under hypoxic culture condition (1% O_2_), MAP17 expression in MHCC-97H and Huh7 cells in the presence or absence of HIF1α knockdown was analyzed by real-time qPCR (G, n = 3) and western blotting (**h**), respectively. **i** Chromatin immunoprecipitation-PCR validation of the regulatory role of HIF-1α in MAP17 expression. Input and ChIP cycle threshold (Ct) values were normalized separately to control as 1. *P < 0.05; **P < 0.01; ***P < 0.001

To determine the critical regulators involved in the hypoxic tumor microenvironment and tumor glycolysis, we performed an integrated analysis by leveraging molecular profiles from TCGA cohort. Based on the hypoxia-related gene signature reported previously [25], we used a dichotomy method (hypoxia-high vs. hypoxia-low) and identified 742 differentially expressed mRNAs associated with hypoxia. Likewise, 285 glycolysis-related genes were also found in HCC. Among these genes, 4 genes (LOXL2, MAP17, LDHA, and EDIL3) were highly expressed in HCC (Fig. 1a). Given the roles and mechanisms of LOXL2, LDHA, and EDIL3 have been well documented in HCC [26–28], MAP17 is selected for further investigation. MAP17 is almost not expressed in normal human tissues except for the kidney (Supplementary Figure 1). To confirm the expression pattern of MAP17 in HCC, we analyzed its expression in a tissue microarray containing 202 pathologist-certified HCC samples by immunohistochemical method. As shown in Fig. 1b, MAP17 immunoreactivity was largely present at the tumor cell membrane and merely distributed in nontumor tissues. Kaplan-Meier curve revealed that HCC patients with higher MAP17 levels had a significantly reduced overall survival (Fig. 1c). By real-time qPCR and western blotting analysis, we noticed that SMMC-7721 and HCC-LM3 cells had a higher endogenous level of MAP17 (Fig. 1d). To certify whether MAP17 is induced by hypoxia in HCC, we cultured three HCC cell lines (SK-H929, MHCC-97H, and Huh7) under both normoxic and hypoxic conditions. As a result, MAP17 expression was significantly boosted by hypoxia at both the mRNA (Fig. 1e) and protein levels (Fig. 1f and Supplementary Figure 2), indicating a regulatory role of HIF1α in MAP17 expression. As the next line of evidence, genetic silencing of HIF1α largely attenuated hypoxia-induced MAP17 expression (Fig. 1g-h). Finally, chromatin immunoprecipitation data showed that HIF1α interacted directly with MAP17 gene promoters under hypoxia (Fig. 1i). Therefore, highly expressed MAP17 may be induced by hypoxia in HCC. In addition to HCC, MAP17 was also overexpressed in many other types of tumors, such as cervical cancer, colorectal cancer, lung cancer, ovarian cancer, pancreatic cancer, gastric cancer, thyroid cancer, uterine corpus endometrial carcinoma, and uterine carcinosarcoma (Supplementary Figure 3), suggesting that dysregulation of MAP17 is a universal phenomenon in human cancers.

### MAP17 promotes the Warburg effect in HCC cells

**Fig. 2:**
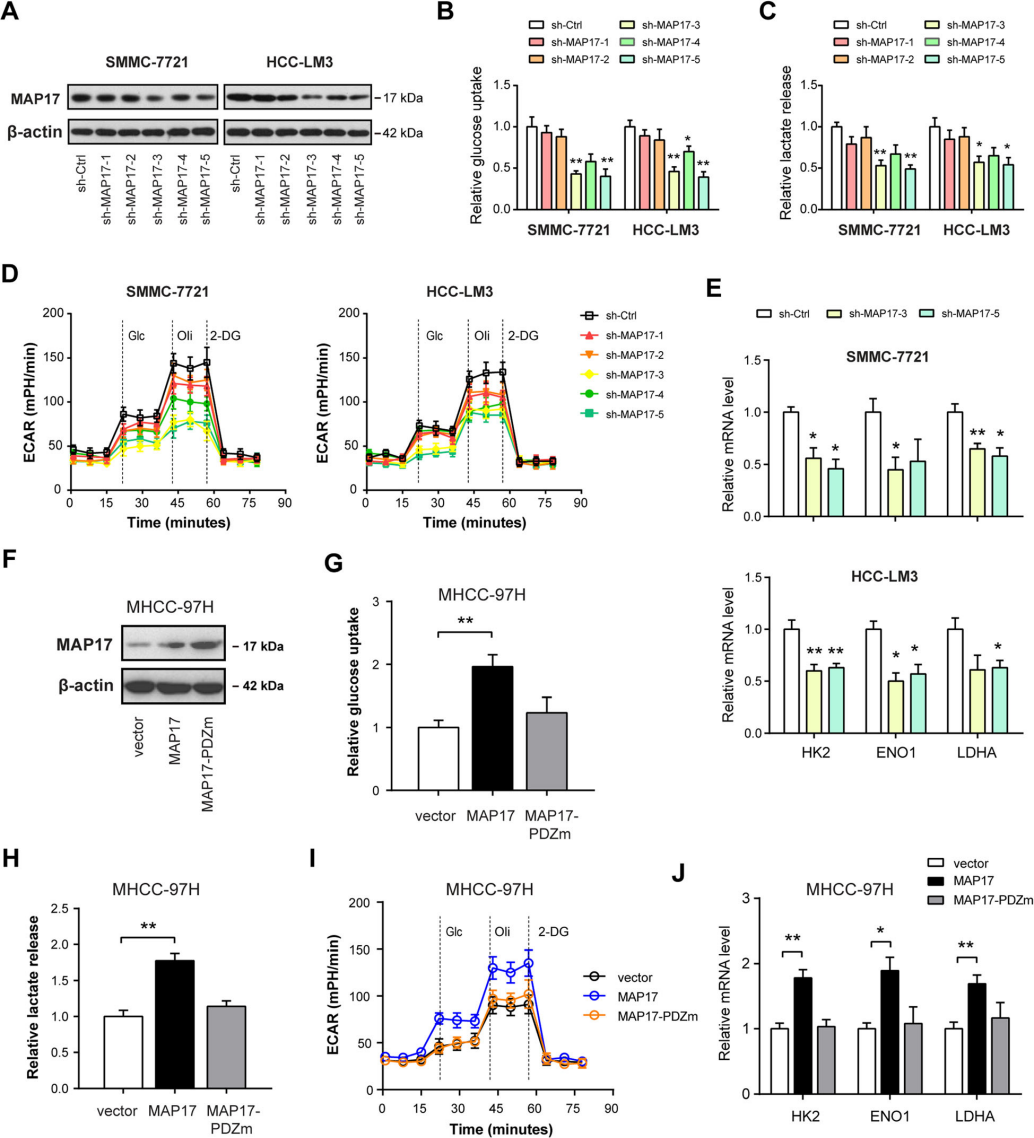
MAP17 promotes the Warburg effect in HCC cells. **a** The knockdown efficiency of 5 independent shRNAs against MAP17 in SMCC-7721 and HCC-LM3 cells was verified by western blotting analysis. **b**-**e** Effect of MAP17 knockdown on the glucose uptake (**b**, n = 3), lactate release (**c**, n = 3), extracellular acidification rate (**d**, n = 3), and expression of glycolytic genes (HK2, ENO1, and LDHA; **e**, n = 3) in SMCC-7721 and HCC-LM3 cells. **f** The overexpression efficiency of MAP17 and its PDZ binding domain mutant MAP17-PDZm in MHCC-97H cells was confirmed by western blotting analysis. **g**-**j** Effect of MAP17 overexpression on the glucose uptake (**g**, n = 3), lactate release (**h**, n = 3), extracellular acidification rate (**i**, n = 3), and expression of glycolytic genes (**j**, n = 3) in MHCC-97H cells. *P < 0.05 and **P < 0.01

To demonstrate whether MAP17 plays a role in HCC glycolysis, in vitro loss-of-function studies were first carried out. Five independent shRNAs against MAP17 were used and two shRNAs (sh-MAP17-#3 and sh-MAP17-#5) led to a > 60% reduction of MAP17 protein expression in both SMMC-7721 and HCC-LM3 cells (Fig. 2a). Consistent with the knockdown efficiency, reduction in MAP17 expression led to a significant decrease in glucose uptake (Fig. 2b), lactate production (Fig. 2c), extracellular acidification rate (Fig. 2d), and expression of glycolytic genes (Fig. 2e) in SMMC-7721 and HCC-LM3 cells. To further confirm the potential regulatory role of MAP17 in HCC glycolysis, we overexpressed MAP17 and its PDZ binding domain mutant MAP17-PDZm in MHCC-97H cells (Fig. 2f). Interestingly, ectopic expression of MAP17 but not MAP17-PDZm enhanced the Warburg effect as evidenced by glucose uptake, lactate release, ECAR, and expression of glycolytic genes (Fig. 2g-j), indicating that the PDZ binding domain of MAP17 plays a key role in its glycolysis-regulating activity.

### MAP17 is required for tumor growth in HCC

**Fig. 3:**
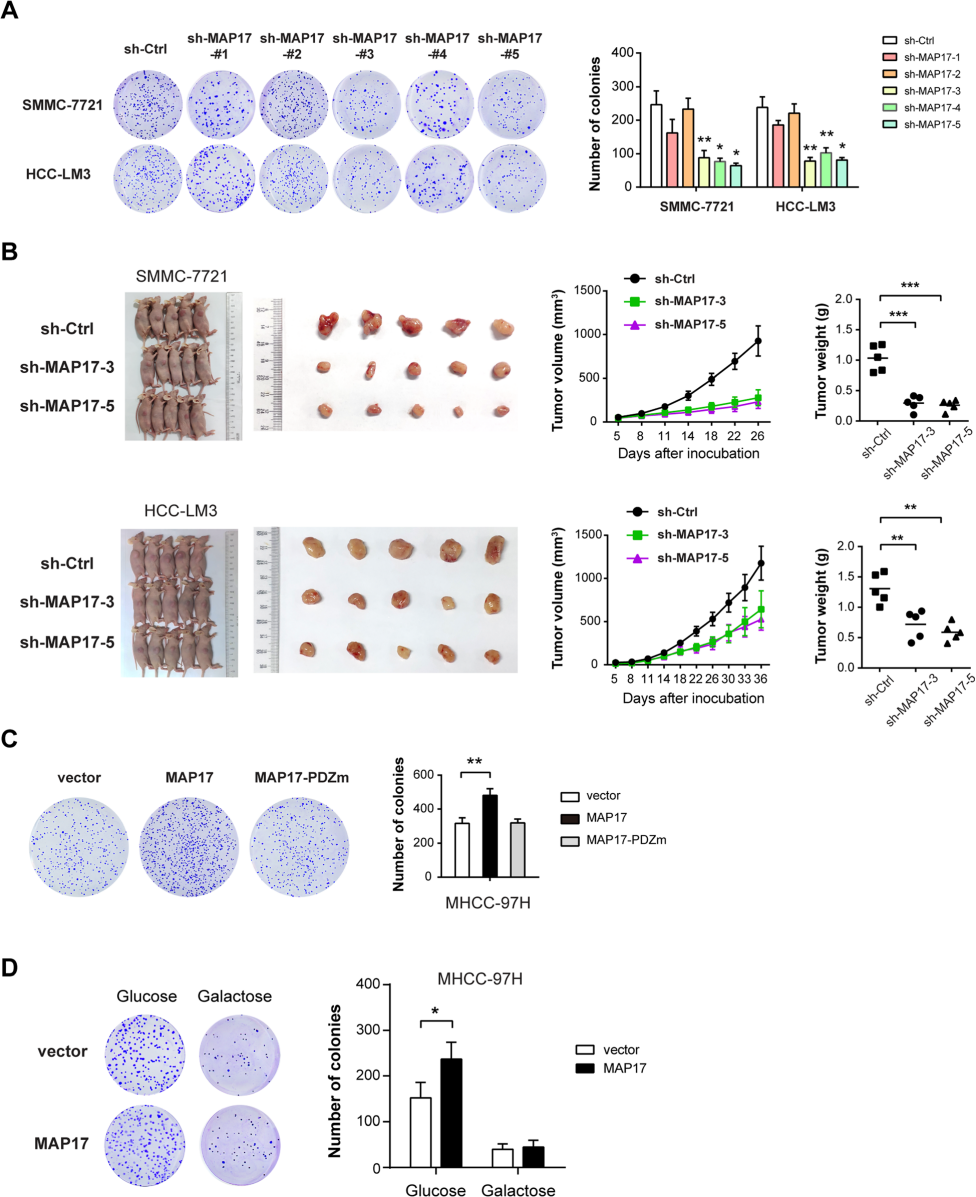
MAP17 is required for tumor growth in HCC. **a** The effect of MAP17 knockdown on in vitro proliferation of SMCC-7721 and HCC-LM3 cells was analyzed by colony formation assay (n = 3). **b** sh-Ctrl and sh-MAP17 SMCC-7721 and HCC-LM3 cells were subcutaneously injected into the nude mice (*n* = 5 per group); gross xenografts, tumor growth curve, and tumor weight were shown. **c** The effect of MAP17 or MAP17-PDZm overexpression on the in vitro proliferation of MHCC-97H cells was analyzed by colony formation assay (n = 3). **d** MHCC-97H cells were cultured in a medium containing galactose (5 mM) but no glucose; the effect of MAP17 overexpression on colony formation ability was further analyzed. *P < 0.05; **P < 0.01; ***P < 0.001

Next, we studied the potential oncogenic roles of MAP17 in HCC. By plate colony formation assay, we found that MAP17 knockdown contributed to decreased proliferative capabilities in SMMC-7721 and HCC-LM3 cells (Fig. 3a). In the subcutaneous xenograft model, MAP17 knockdown remarkably retarded tumor growth as demonstrated by tumor volume, tumor weight, and the expression of proliferation index Ki67 (Fig. 3b and Supplementary Figure 4). In contrast, MAP17 but not MAP17-PDZm overexpression enhanced the colony formation ability of MHCC-97H cells (Fig. 3c), suggesting that MAP17-mediated increase in tumorigenesis is also dependent on its PDZ-binding domain. To determine whether MAP17-dependent tumorigenesis is coupled to glycolysis, we replaced glucose in the culture medium with galactose, which occurs at a much lower rate than glucose entry into glycolysis and thereby suppressing glycolytic flux. As shown in Fig. 3d, galactose largely compromised the growth-promoting role induced by MAP17. Collectively, these findings above indicate that MAP17 may coordinate the Warburg effect to promote tumor growth in HCC.

### MAP17 regulates intracellular ROS to enhance the tumorigenic and glycolytic phenotypes

**Fig. 4:**
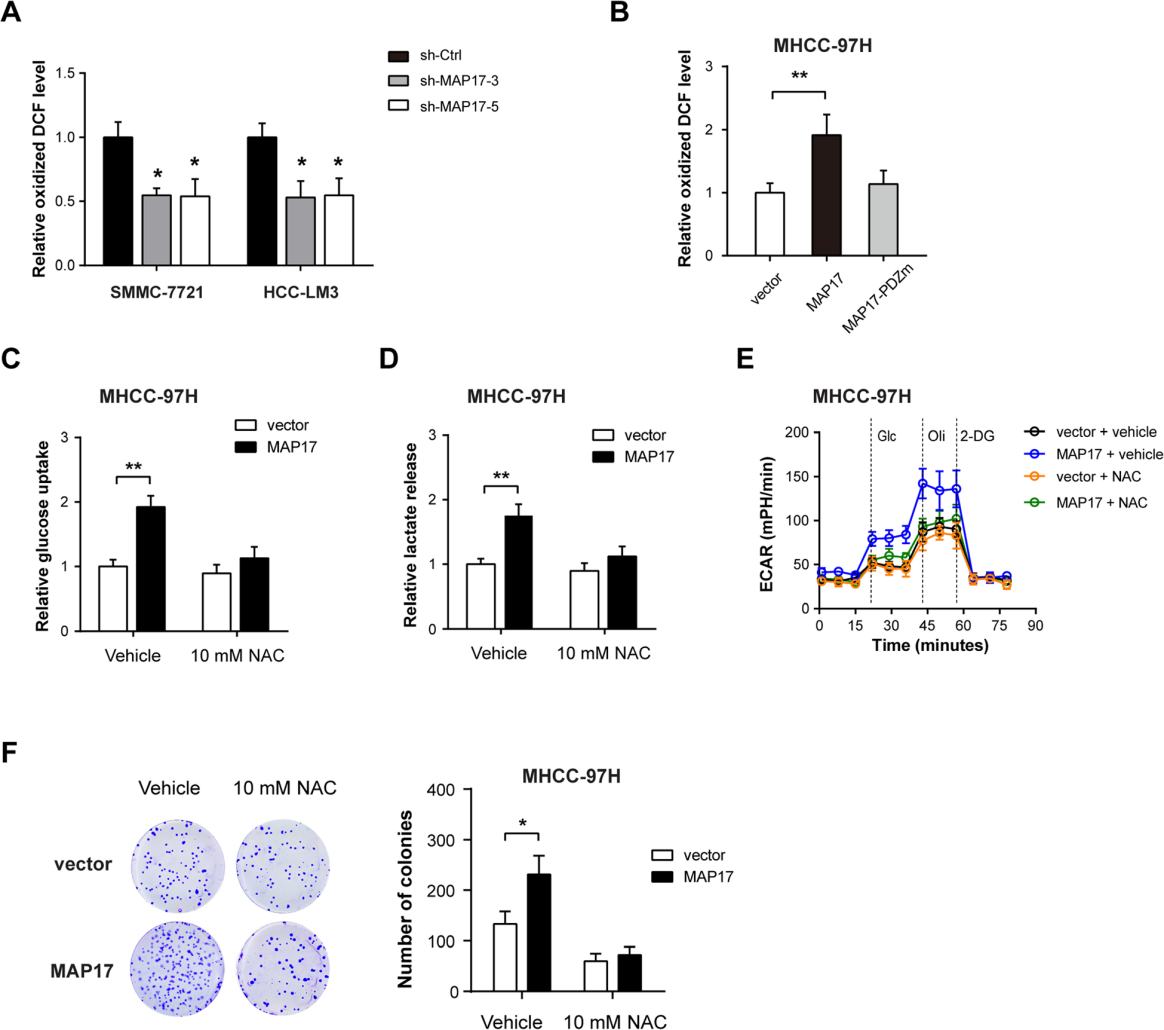
MAP17 regulates intracellular ROS to enhance the tumorigenic and glycolytic phenotypes. **a** The effect of MAP17 knockdown on ROS generation in SMCC-7721 and HCC-LM3 cells. **b** The effect of MAP17 or MAP17-PDZm overexpression on ROS generation in MHCC-97H cells. **c**-**e** Effects of MAP17 overexpression on glucose uptake (**c**), lactate release (**d**), and ECAR (**e**) were analyzed in the presence or absence of 10 mM N-acetyl cysteine (NAC) treatment. **f** The effect of MAP17 overexpression on cell proliferation of MHCC-97H cells in the presence or absence of 10 mM N-acetyl cysteine (NAC) treatment was determined by plate colony formation assay. *P < 0.05 and **P < 0.01

It is well known that MAP17 interacts with PDZK1 and other members of the NHeRF (sodium hydrogen exchange regulatory factor) family [15, 29]. Through proximity ligation assay and co-immunoprecipitation assay, we observed a physical interaction between MAP17 and PDZK1 in SMMC-7721 and HCC-LM3 cells (Supplementary Figure 5A-B). Interaction between MAP17 and PDZK1 potentiates SGLT1/2-mediated glucose uptake in a sodium-dependent manner in *Xenopus* oocytes and mammary cells [16, 30]. However, since SGLT1/2 expression was not present in HCC tissues (Supplementary Figure 5C), we ruled out the possibility of its role in MAP17-induced glycolysis. ROS is a key mediator in many signaling cascades that are related to cell proliferation and transformation [31]. Although MAP17 has no enzymatic activity, its overexpression can lead to an increase of 30–40% in ROS generation [32]. Indeed, we found that MAP17 knockdown resulted in a ~ 40% reduction of ROS in SMMC-7721 and HCC-LM3 cells (Fig. 4a), while MAP17 overexpression increased ROS level in MHCC-97H cells (Fig. 4b). To test whether ROS is responsible for the enhanced tumorigenic properties of MAP17 in HCC, we blocked ROS with the antioxidant N-acetylcysteine (NAC). In MHCC-97H cells overexpressing MAP17, NAC treatment significantly reduced the glycolytic phenotypes of MAP17 (Fig. 4c-e). In addition, the number of colonies of MAP17-expressing cells was also decreased by NAC (Fig. 4f).

### AKT and HIF1α are the downstream effectors of ROS in HCC

**Fig. 5:**
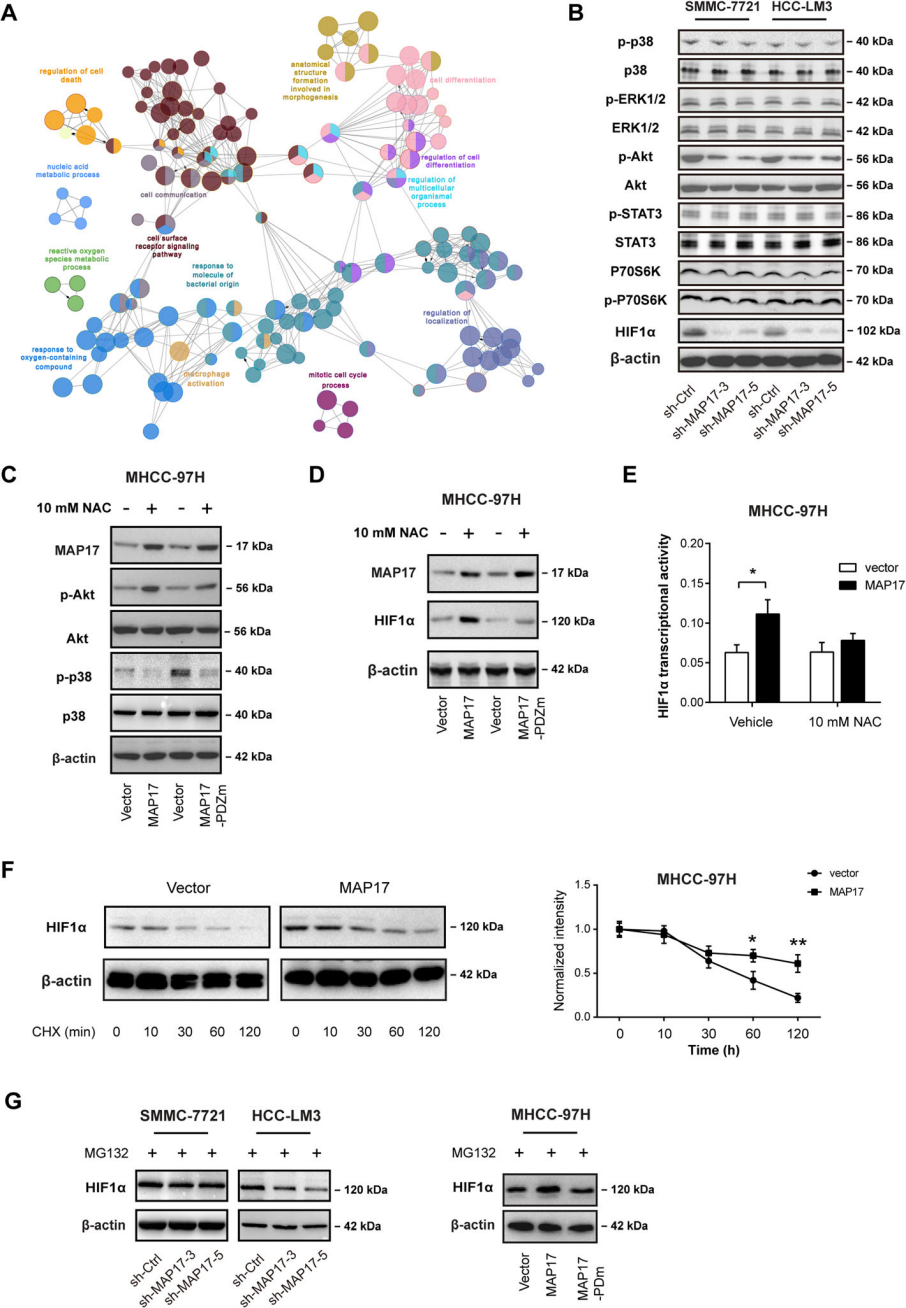
AKT and HIF1α are the downstream effectors of ROS in HCC. **a** RNA sequencing analysis of the transcriptional changes induced by MAP17 knockdown in HCC-LM3 cells; ClueGO analysis of the differentially expressed genes by CytoScape software. **b** The effect of MAP17 knockdown on the activity of ROS-associated signaling pathways (p38 MAPK, ERK1/2, AKT, STAT3, P70S6K, and HIF1α) in SMCC-7721 and HCC-LM3 cells was analyzed by western blotting. **c** The effect of MAP17 or MAP17-PDZm overexpression on AKT activity in the presence or absence of 10 mM N-acetyl cysteine (NAC) treatment was determined by western blotting. **d** Western blotting analysis of MAP17 or MAP17-PDZm overexpression on HIF1α protein in the presence or absence of 10 mM N-acetyl cysteine (NAC) treatment. **e** The effect of MAP17 or MAP17-PDZm overexpression on HIF1α transcriptional activity in MHCC-97H cells. **f** Western blotting analysis of HIF1α protein level in MAP17-overexprssing or vector control cells at indicated times upon 20 μg/ml CHX treatment. **g** Western blotting analysis of HIF1α protein level in MAP17 knockdown or overexpression cells treated with 10 μM MG132 under hypoxic conditions. *P < 0.05 and **P < 0.01

To pursue the cellular mechanism of MAP17, we performed RNA sequencing analysis in sh-Ctrl and sh-MAP17 HCC-LM3 cells. The result showed that a total of 1909 genes whose expressions were downregulated in response to MAP17 knockdown in HCC-LM3 cells. Interestingly, functional enrichment analysis of these genes demonstrated significant enrichment of ROS metabolic process, cell surface receptor signaling, cell communication, mitotic cell cycle progression, and regulation of cell differentiation (Fig. 5a). ROS act as a second messenger can activate many signaling pathways, such as p42/p44 mitogen-activated protein kinase (MAPK), p38 MAPK, signal transducers and activators of transcription (STAT), Akt/protein kinase B, p70S6K, and HIF1α [32]. By western blotting analysis, we found that MAP17 knockdown significantly inhibited the activation of AKT and HIF1α but not p42/p44, p38, STAT3, and p70S6K in SMMC-7721 and HCC-LM3 cells (Fig. 5b). Conversely, MAP17 overexpression enhanced AKT activation, which was downregulated by the addition of 10 mM NAC (Fig. 5c). The real-time qPCR results showed that genetic manipulation of MAP17 failed to affect HIF1α mRNA expression in HCC cells (Supplementary Figure 6). However, HIF1α protein level (Fig. 5d) and transcriptional activity (Fig. 5e) were significantly enhanced by MAP17 overexpression and further blocked by neutralization of ROS with NAC, suggesting that MAP17 may exert a posttranslational regulatory role on HIF1α protein stability. Indeed, overexpression of MAP17 reduced the degradation rate of HIF1α protein in MHCC-97H cells (Fig. 5f). Moreover, cells used for MAP17 knockdown or overexpression were pretreated with MG132 to prevent HIF1α from being degraded under hypoxic conditions. As a result, significantly less HIF1α was accumulated during MG132 treatment following MAP17 knockdown; in contrast, more HIF1α was accumulated following MAP17 overexpression (Fig. 5g). To confirm whether AKT and HIF1α mediate the roles of MAP17 in HCC, a specific inhibitor of AKT (MK-2206) and specific siRNAs against HIF1α were used in MHCC-97H cells. Expectedly, MAP17-dependent glycolysis and tumor growth were effectively blocked by MK-2206 (Supplementary Figure 7) or HIF1α knockdown (Supplementary Figure 8). Taken together, AKT and HIF1α may act as the downstream effectors of the MAP17/ROS axis in HCC.

### MAP17 correlates p-AKT and HIF1α in clinical HCC samples

**Fig. 6:**
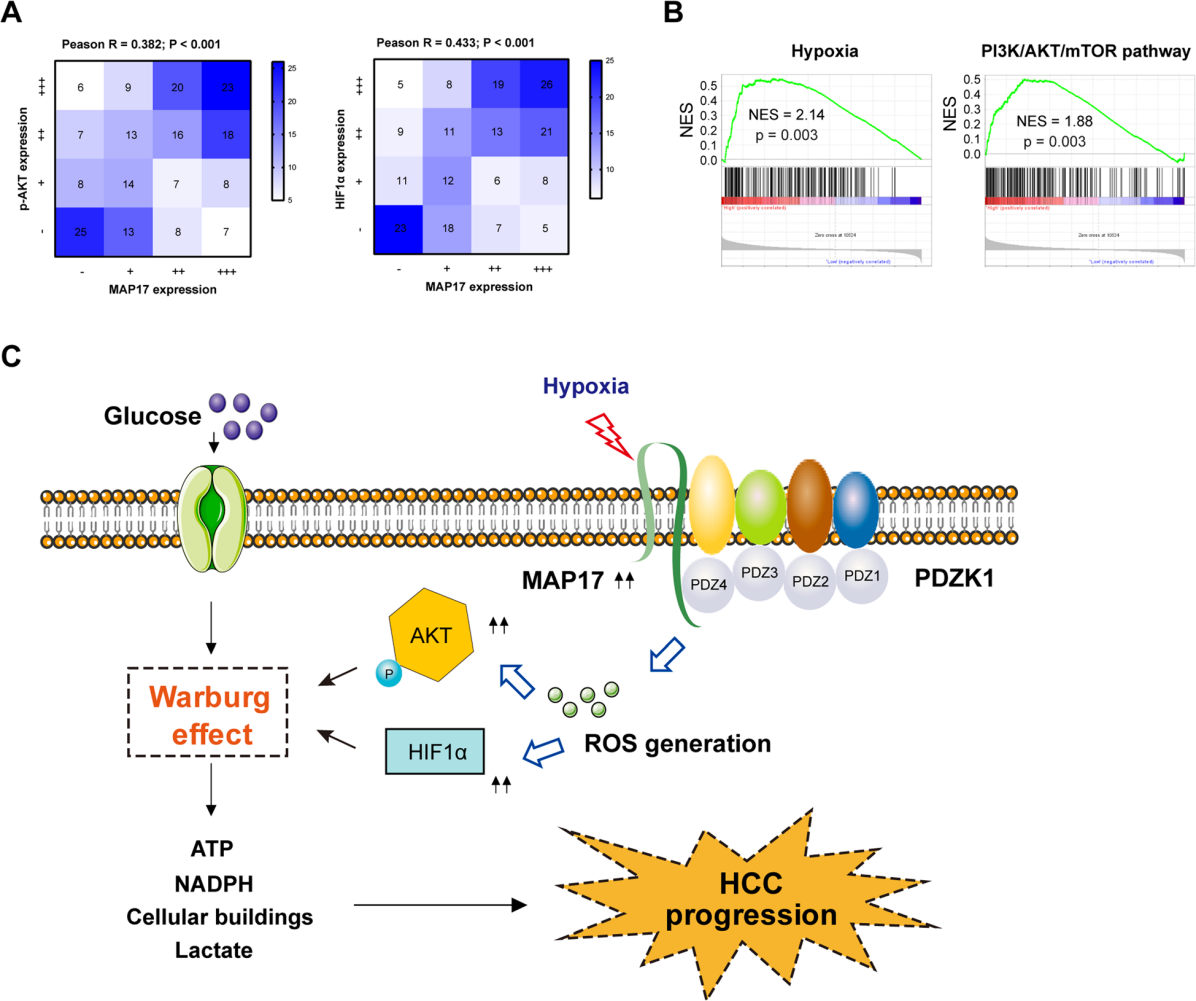
MAP17 correlates p-AKT and HIF1α in clinical HCC samples. **a** Correlation between MAP17 expression and HIF1α or p-AKT level in human HCC tissues (*n* = 202). **b** Gene set enrichment analysis of gene sets related to MAP17 expression in TCGA HCC cohort; NES, normalized enrichment score; false discovery rate (FDR) was set at 0.25. **c** Schematic diagram for MAP/ROS-mediated metabolic modes and tumor growth in HCC

Finally, we determined the expression of p-AKT and HIF1α by IHC in a cohort of 202 HCC patients. As a result, there was a significantly positive correlation between MAP17 expression and p-AKT (Pearson R = 0.382; *P* < 0.001) or HIF1α expression (Pearson R = 0.433; P < 0.001) in the HCC samples (Fig. 6a). Moreover, we tested TCGA cohort and found that MAP17 expression was closely associated with hypoxia gene signature and PI3K/AKT pathway (Fig. 6b). These findings further confirm the molecular mechanism of MAP17 in the clinical setting.

## Discussion

Metabolic reprogramming in the tumor microenvironment is commonly seen in a wide spectrum of human solid cancers and emerged as a central contributor to the tumor progression machinery. Dysregulated growth factor signaling, activation of HIF-1α-transcription, activation of oncogenes or loss-of-function of suppressor genes contributes to cancer metabolic reprogramming. In the present study, our integrated analysis for the first time identified MAP17 as a major glycolytic regulator in response to hypoxic tumor microenvironment. Through regulation of AKT activation and HIF1α, MAP17 facilitates the Warburg effect to promote tumor growth in HCC (Fig. 6c).

In normal human tissues, MAP17 is dominantly restricted to epithelial cells from the kidney. However, MAP17 overexpression has been reported in many tumor types, such as lung cancer, breast cancer, laryngeal cancer, rectal cancer, and prostate cancer [19, 33–35]. Notably, increased MAP17 expression was present at advanced or metastatic tumors and associated with tumor progression. In HCC, MAP17 expression is positively correlated with gender, distant metastasis, early recurrence, and serum alpha-fetoprotein [36]. Consistent with this finding, we confirmed that higher MAP17 expression is associated with a poor prognosis, indicating that MAP17 may be a novel prognostic biomarker for HCC patients. In normal human epidermal keratinocytes, MAP17 is upregulated in response to interferon-gamma, interleukin 4 (IL-4), IL-6, IL-17A, or IL-22 [37]. However, little is known about the reason for dysregulated MAP17 in cancers. Here, our findings explained that hypoxia acts as an inducer for MAP17 expression in HCC. MAP17 plays pleiotropic oncogenic roles in several human cancers, such as cell apoptosis [38], cell invasion [36], tumor growth [20, 39], chemotherapy sensitivity [35, 40, 41], epithelial mesenchymal transition (EMT) [42], stem cell-like properties [17], and inflammatory response [14, 18]. In this study, we presented a novel regulatory role of MAP17 in the Warburg effect, which further broadens the current knowledge of the hypoxia-induced changes in metabolism during HCC development. Notably, increasing evidence has shown that metabolic reprogramming other than Warburg effect is also essential for tumor development and progression. Indeed, other metabolic features, in particular, the reverse Warburg effect, metabolic symbiosis, and addiction to glutamine metabolism, create challenges for anti-cancer treatment due to adaptive or acquired chemoresistance. Therefore, a comprehensive understanding of metabolic reprogramming may facilitate the development of promising therapeutic strategies by repositioning drug (such as metformin) targeting metabolic reprogramming [43].

The C-terminal sequence of MAP17 contains a PDZ-binding domain, which allows its interaction with PDZ domain-containing proteins, especially PDZK1. Specifically, the MAP17 PDZ-binding domain is essential for its oncogenic activities. For instance, disruption of this sequence of MAP17 by point mutations abolishes its ability to promote the tumorigenic capability of malignant melanoma cells [20]. In line with this observation, we found that ectopic expression of PDZ binding domain mutant MAP17-PDZm cannot promote the Warburg effect or play a growth-promoting effect in HCC. The interaction of MAP17 with SGLT1/2 proteins can increase glucose uptake to enhance glycolytic flux [16, 30]. Interestingly, we revealed that MAP17-induced glycolysis is not dependent on SGLT1/2 in HCC. MAP17 has been reported to activate AKT/mTOR pathway in HCC cells [36]. However, the detailed molecular mechanism remains unclear. Accumulated evidence suggests that the tumorigenic roles of MAP17 are closely associated with an increase in ROS generation. Indeed, our results supported that ROS is generated in a MAP17-dependent manner and is responsible for MAP17-mediated glycolytic and oncogenic activities. Indeed, ROS removal by treatment of antioxidant NAC largely compromised the tumorigenic roles of MAP17. ROS might act as an intracellular signal or a second messenger to initiate downstream cascades that induce malignant phenotypes in cancers. In this study, we revealed that MAP17 triggers a ROS-dependent AKT and HIF1α activation. AKT can modulate aerobic glycolysis through multiple pathways, including but not limited to activation of HIF1α, mTOR, c-Myc, and the subsequent expression of glycolytic enzymes [44]. HIF1α is a well-known transcriptional factor for the Warburg effect [45]. Thus, it is reasonable that AKT and HIF1α are functional mediators of MAP17 in HCC. Although we uncovered a mechanism of MAP17-dependent glycolysis in HCC, the reason for how ROS is increased by MAP17 is not known and warrants further investigations. One possibility is that a direct interaction with the membrane transporters, changing the intracellular redox level through altering the intra/extracellular ion balance [32]. In addition, MAP17 can activate the Notch pathway to promote stem cell-like properties through direct interaction with NUMB via the PDZ-binding domain [17]. Therefore, we cannot rule out other possibilities that are responsible for MAP17-dependent glycolysis in HCC.

## Conclusions

We report here that the hypoxic tumor microenvironment may induce MAP17 expression in HCC. Moreover, we elucidate that MAP17-induced ROS activate AKT and increase HIF1α protein stability to promote the Warburg effect and tumor growth. Therefore, MAP17 could function as a glycolysis enhancer in HCC, and targeting the MAP17/ROS axis may represent a new method for the treatment of HCC.

## Supplementary Information

**Additional file 1: Supplementary Figure 1.** Expression pattern of MAP17 in normal human tissues and cells. **Supplementary Figure 2.** Expression of MAP17 under hypoxic condition in HCC cells. **Supplementary Figure 3.** Expression pattern of MAP17 in human cancers. **Supplementary Figure 4.** MAP17 knockdown inhibits tumor growth in vivo. **Supplementary Figure 5.** Interaction between MAP17 and PDZK1 in HCC cells. **Supplementary Figure 6.** The effect of MAP17 knockdown or overexpression on HIF1α mRNA expression in HCC. **Supplementary Figure 7.** MK-2206 blocks the tumorigenic and glycolytic phenotypes induced by MAP17. **Supplementary Figure 8.** HIF1α knockdown blocks the tumorigenic and glycolytic phenotypes induced by MAP17.

## Data Availability

All data generated or analyzed during this research are included in this manuscript.

## Abbreviations

HCC: Hepatocellular carcinoma
ROS: Reactive oxygen species
HIF1α: Hypoxia-inducible factor 1α
SGLT1/2: Sodium-dependent glucose transporter 1/2
RT-PCR: Reverse transcription-polymerase chain reaction
FPKM: Fragments Per Kilobase Million
ECAR: Extracellular acidification rate
2-DG: 2-deoxyglucose
NAC: N-acetylcysteine
