# Supplementary material for: Hypoxia-dependent expression of MAP17 coordinates the Warburg effect to tumor growth in hepatocellular carcinoma
Source: J Exp Clin Cancer Res. 2021 Apr 8;40:121. doi: 10.1186/s13046-021-01927-5 (PMC8028089; doi:10.1186/s13046-021-01927-5)
Supplement: Supplementary file 1 — Additional file 1: Supplementary Figure 1. Expression pattern of MAP17 in normal human tissues and cells. Supplementary Figure 2. Expression of MAP17 under hypoxic condition in HCC cells. Supplementary Figure 3. Expression pattern of MAP17 in human cancers. Supplementary Figure 4. MAP17 knockdown inhibits tumor growth in vivo. Supplementary Figure 5. Interaction between MAP17 and PDZK1 in HCC cells. Supplementary Figure 6. The effect of MAP17 knockdown or overexpression on HIF1α mRNA expression in HCC. Supplementary Figure 7. MK-2206 blocks the tumorigenic and glycolytic phenotypes induced by MAP17. Supplementary Figure 8. HIF1α knockdown blocks the tumorigenic and glycolytic phenotypes induced by MAP17. [file 13046_2021_1927_MOESM1_ESM.docx]

**Supplementary Information**

**Supplementary Figure 1**

**
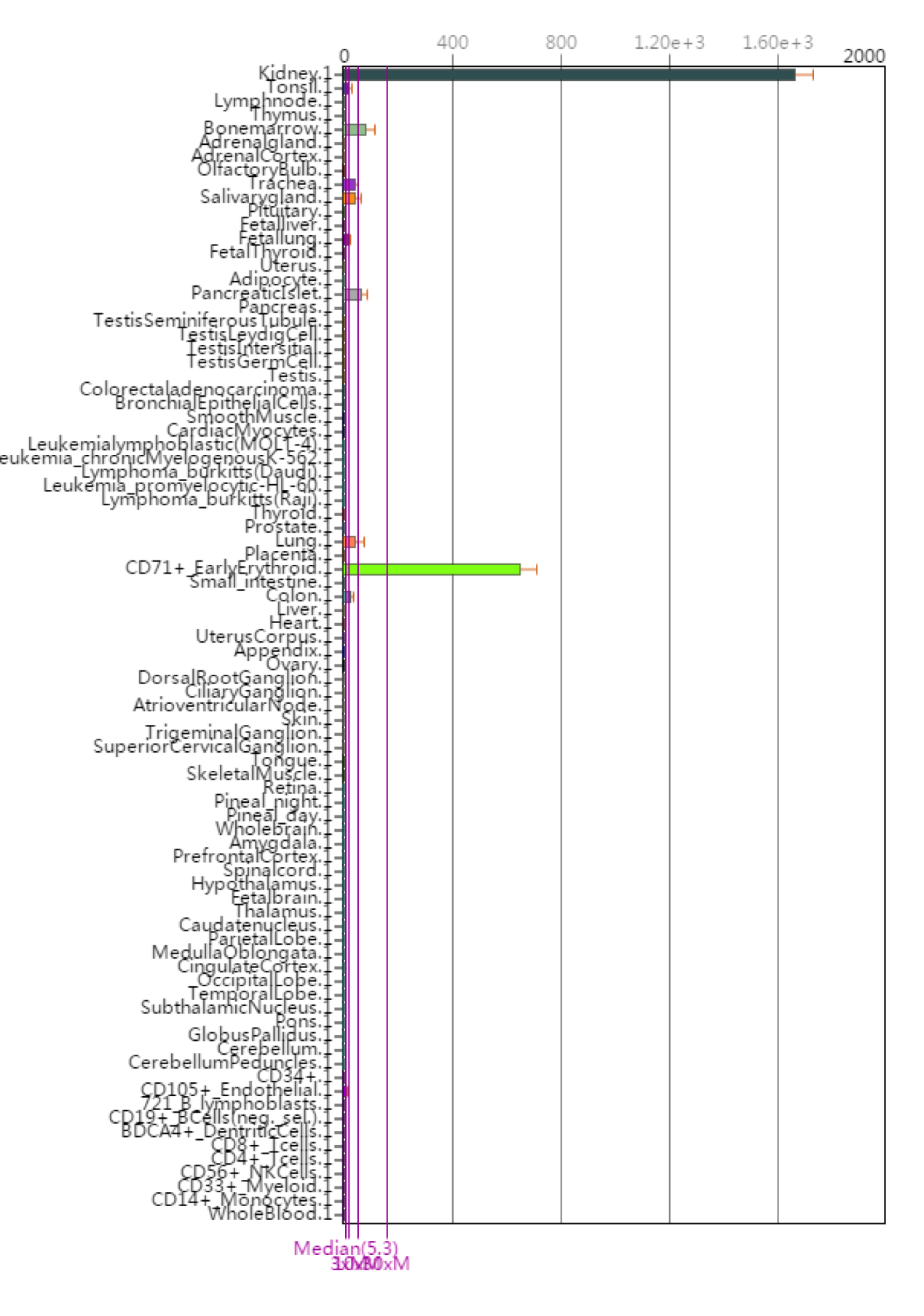
**

**Supplementary Figure 1 Expression pattern of MAP17 in normal human tissues and cells**

Data were acquired from BioGPS (http://biogps.org).

**Supplementary Figure 2**

**
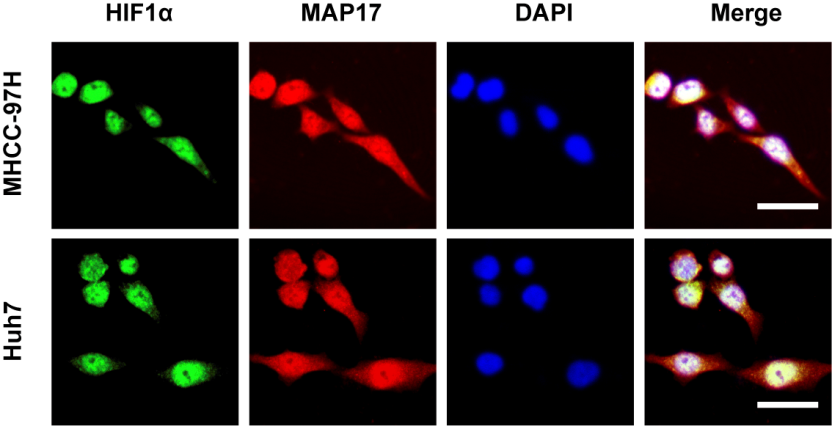
**

**Supplementary Figure 2 Expression of MAP17 under hypoxic condition in HCC cells**

Fluorescent immunostaining analysis of the cellular location of HIF1αand MAP17 in two HCC cell lines (MHCC-97H and Huh7) under hypoxia; scale bar: 10 μm.

**Supplementary Figure 3**

**
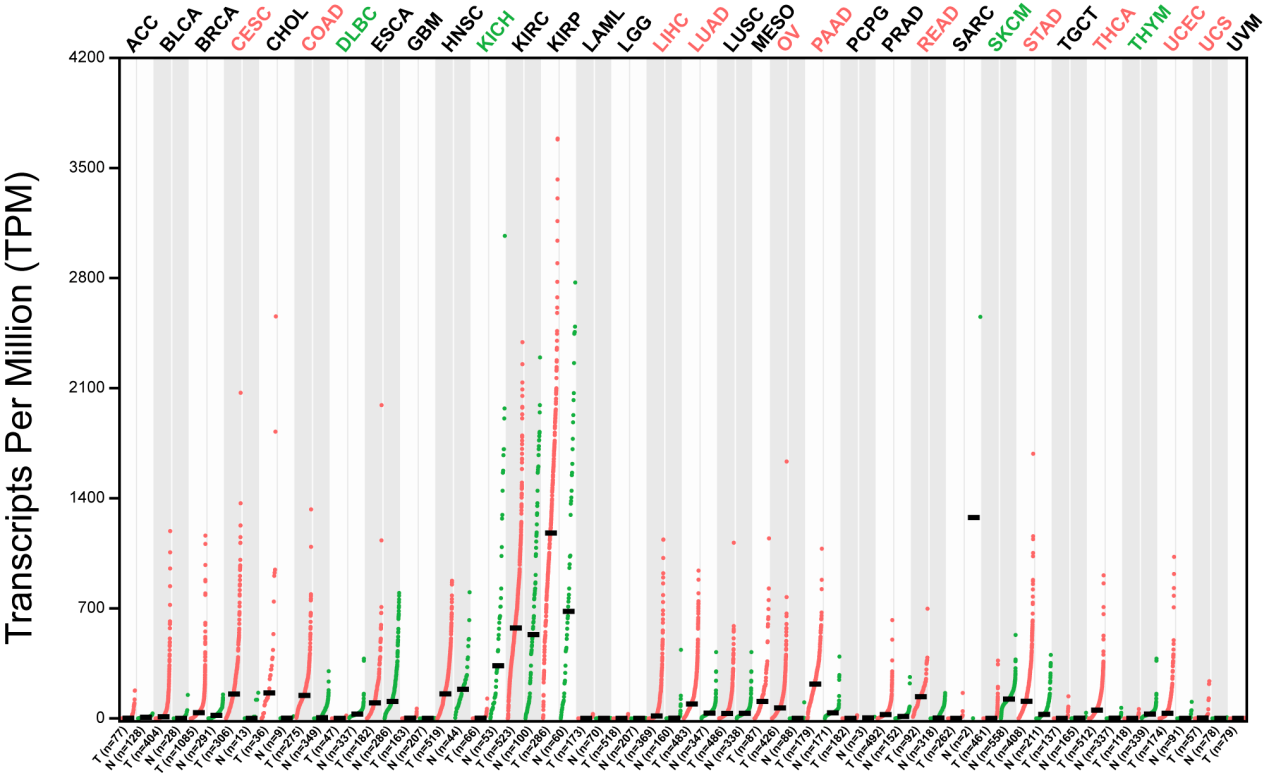
**

**Supplementary Figure 3 Expression pattern of MAP17 in human cancers**

ACC, Adrenocortical carcinoma; BLCA, Bladder urothelial carcinoma; BRCA, Breast invasive carcinoma; CESC, Cervical squamous cell carcinoma and endocervical adenocarcinoma; CHOL, Cholangio carcinoma; COAD, Colon adenocarcinoma; DLBC, Lymphoid neoplasm diffuse large B-cell lymphoma; ESCA, Esophageal carcinoma; GBM, Glioblastoma multiforme; HNSC, Head and neck squamous cell carcinoma; KICH, Kidney chromophobe; KIRC, Kidney renal clear cell carcinoma; KIRP, Kidney renal papillary cell carcinoma; LAML, Acute myeloid leukemia; LGG, Brain lower grade glioma; LIHC, Liver hepatocellular carcinoma; LUAD, Lung adenocarcinoma; LUSC, Lung squamous cell carcinoma; MESO, Mesothelioma; OV, Ovarian serous cystadenocarcinoma; PAAD, Pancreatic adenocarcinoma; PCPG, Pheochromocytoma and paraganglioma; PRAD, Prostate adenocarcinoma; READ, Rectum adenocarcinoma; SARC, Sarcoma; SKCM, Skin cutaneous melanoma; STAD, Stomach adenocarcinoma; TGCT, Testicular germ cell tumors; THCA, Thyroid carcinoma; THYM, Thymoma; UCEC, Uterine corpus endometrial carcinoma; UCS, Uterine carcinosarcoma; UVM, Uveal melanoma.

**Supplementary Figure 4**

**
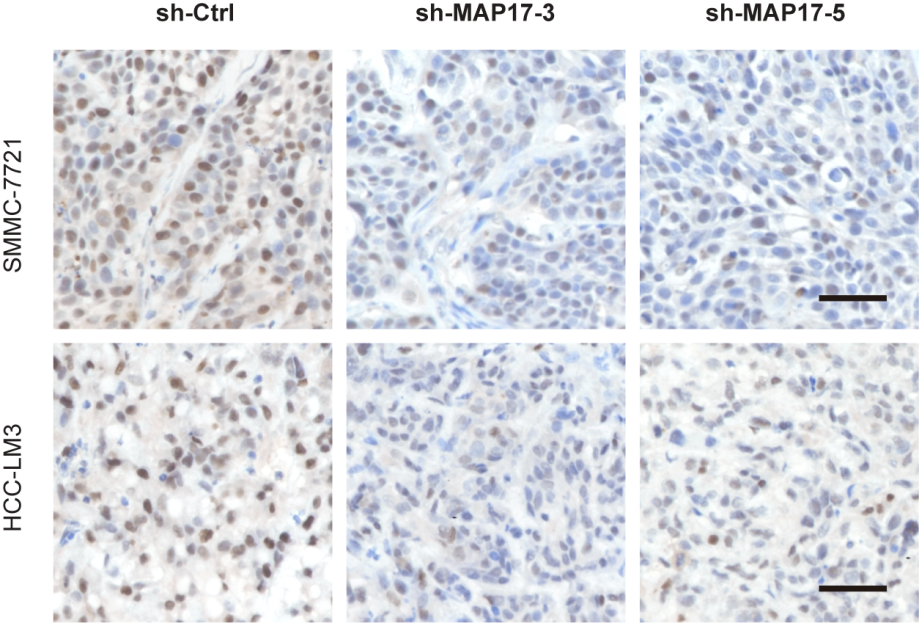
**

**Supplementary Figure 4 MAP17 knockdown inhibits tumor growth *in vivo***

IHC analysis of Ki67 staining in sh-Ctrl and sh-MAP17 from indicated xenograft tumor tissues; scale bar: 50 μm.

**Supplementary Figure 5**

**
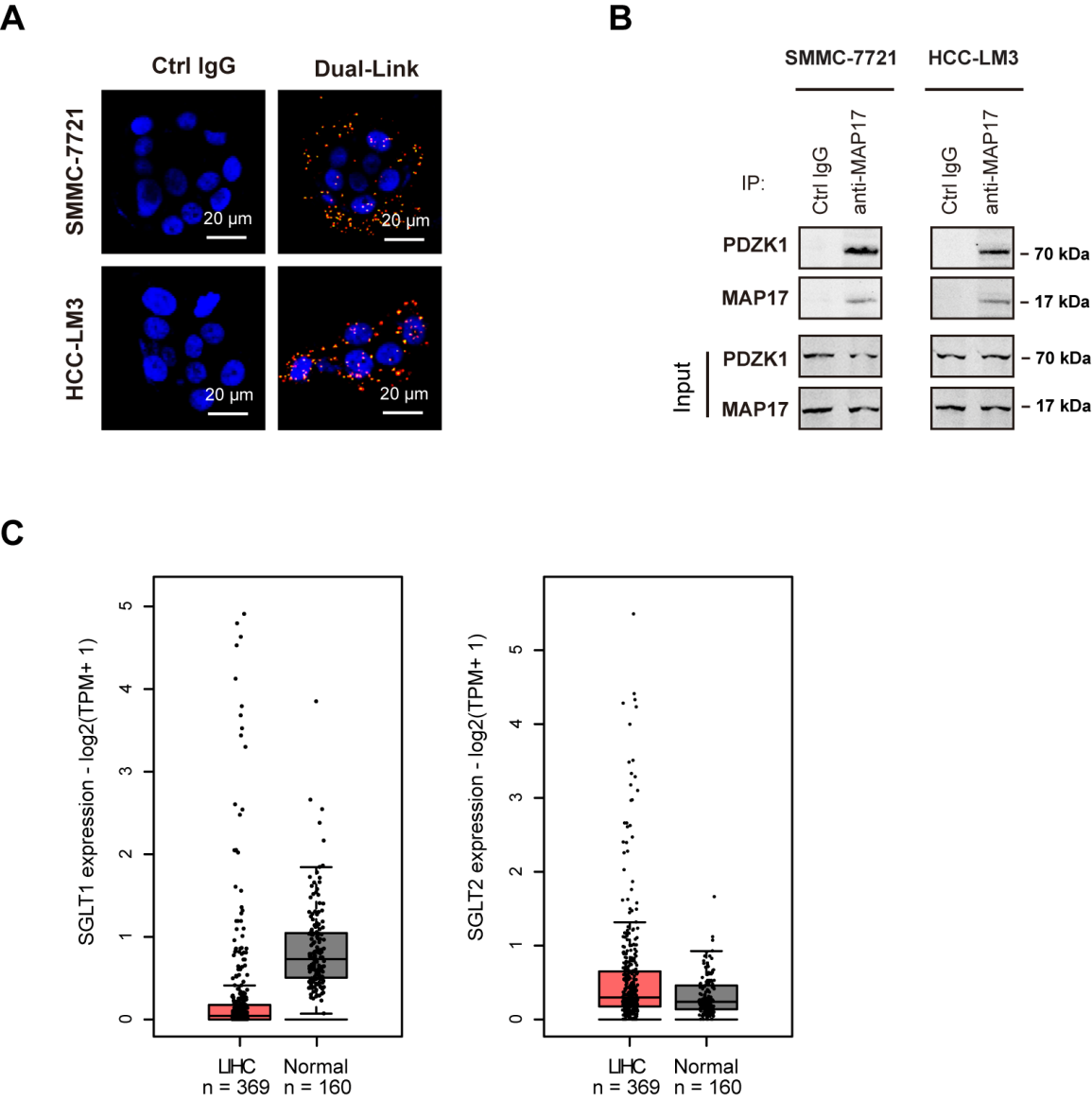
**

**Supplementary Figure 5 Interaction between MAP17 and PDZK1 in HCC cells**

(A) In situ proximity ligation assay analysis of the interaction between MAP17 and PDZK1 in SMCC-7721 and HCC-LM3 cells. (B) Co-immunoprecipitation analysis of the interaction between MAP17 and PDZK1 in SMCC-7721 and HCC-LM3 cells. (C) The expression level of SGLT1 and SGLT2 in liver cancer and normal liver tissues.

**Supplementary Figure 6**


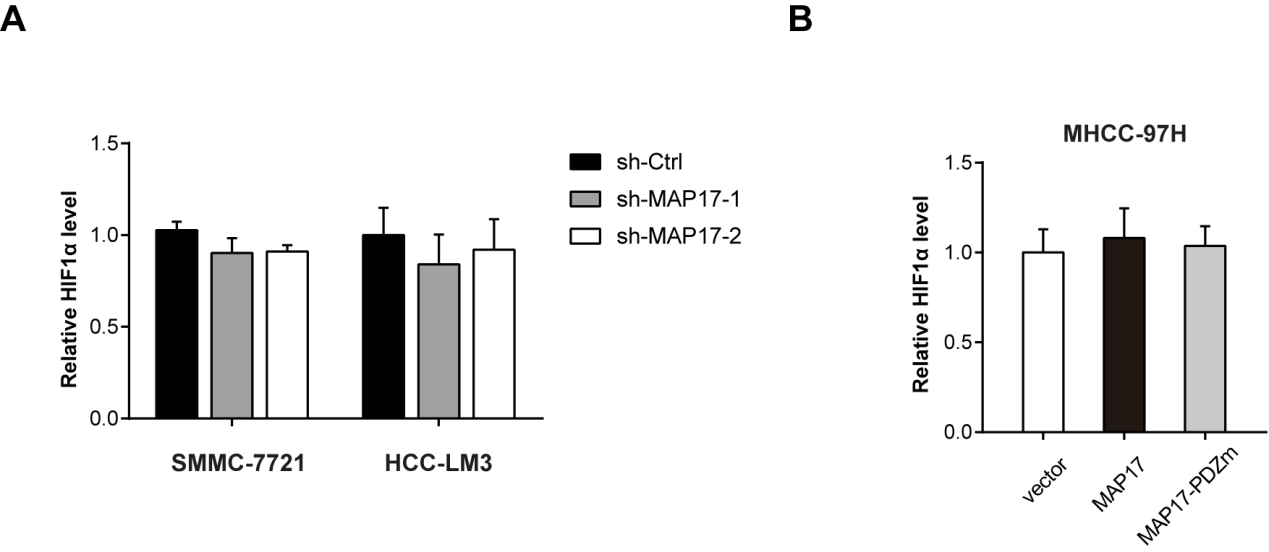


**Supplementary Figure 6 The effect of MAP17 knockdown or overexpression on HIF1α mRNA expression in HCC**

(A) The effect of MAP17 knockdown on HIF1α mRNA expression in SMCC-7721 and HCC-LM3 cells was analyzed by real-time qPCR. (B) The effect of MAP17 overexpression on HIF1α mRNA expression in MHCC-97H cells was analyzed by real-time qPCR.

**Supplementary Figure 7**


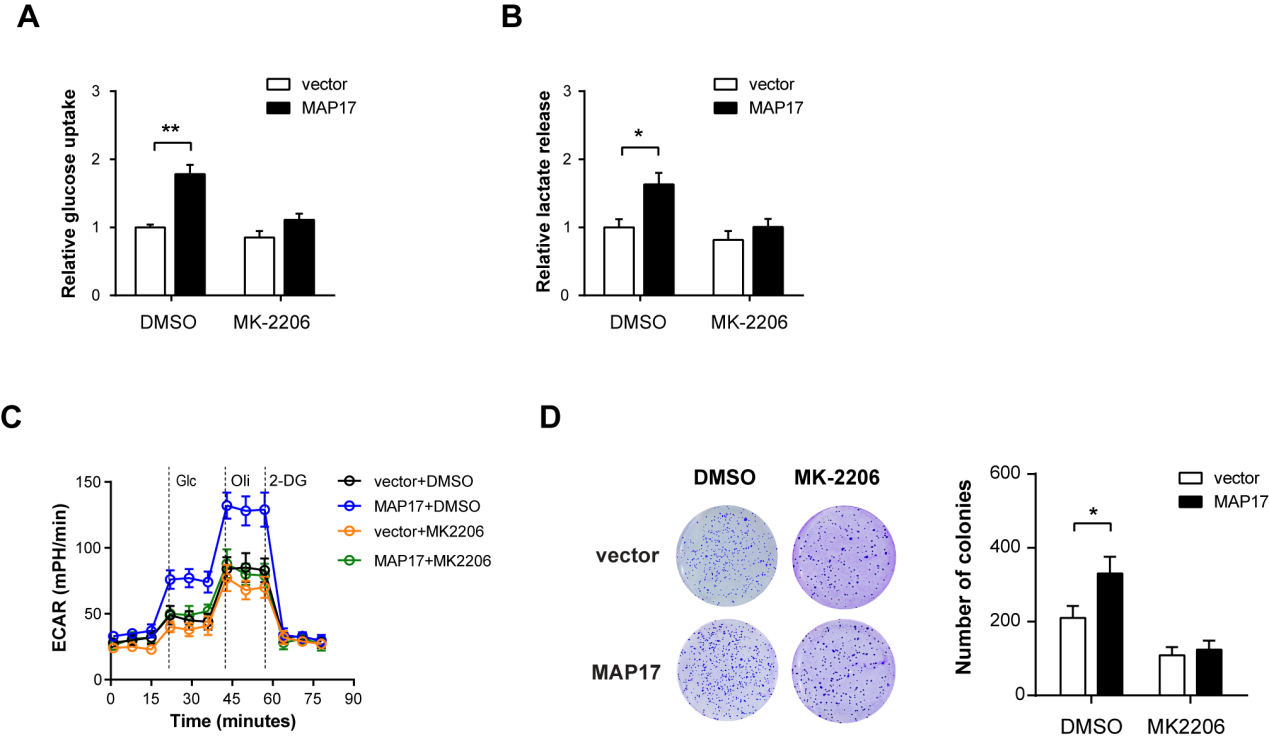


**Supplementary Figure 7 MK-2206 blocks the tumorigenic and glycolytic phenotypes induced by MAP17**

(A-C) Effects of MAP17 overexpression on glucose uptake (A), lactate release (B), and ECAR (C) were analyzed in the presence or absence of treatment with a specific AKT inhibitor (MK-2206, 5 μM) in MHCC-97H cells. (D) The effect of MAP17 overexpression on cell proliferation of MHCC-97H cells in the presence or absence of 5 μM MK-2206 was determined by plate colony formation assay. **P* < 0.05 and ***P* < 0.01.

**Supplementary Figure 8**

**
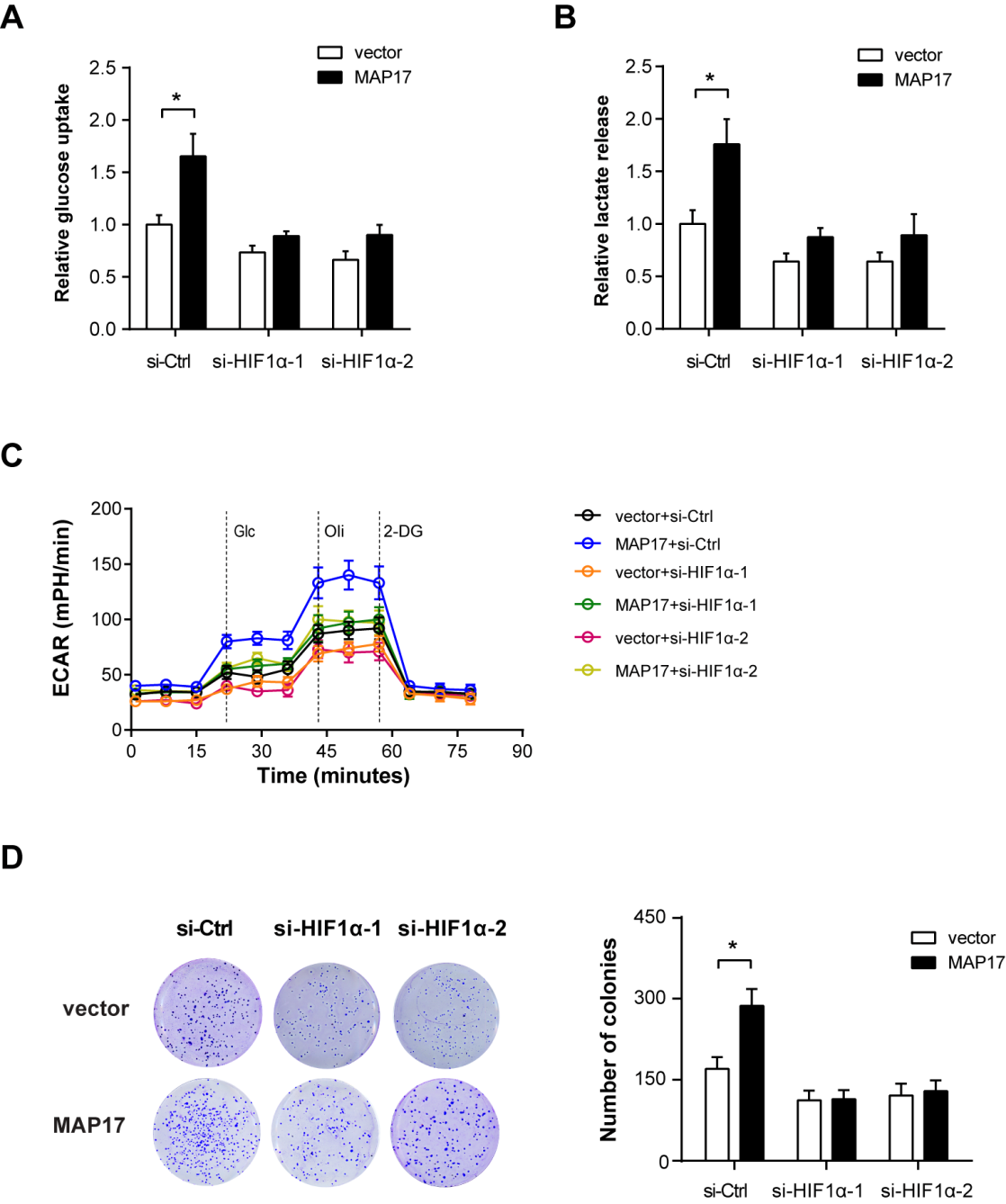
**

**Supplementary Figure 8 HIF1α knockdown blocks the tumorigenic and glycolytic phenotypes induced by MAP17**

(A-C) Effects of MAP17 overexpression on glucose uptake (A), lactate release (B), and ECAR (C) were analyzed in the presence or absence of HIF1α knockdown in MHCC-97H cells. (D) The effect of MAP17 overexpression on cell proliferation of MHCC-97H cells in the presence or absence of HIF1α knockdown was determined by plate colony formation assay. **P* < 0.05.
